# Augmentation of cellular NAD^+^ by NQO1 enzymatic action improves age‐related hearing impairment

**DOI:** 10.1111/acel.13016

**Published:** 2019-07-28

**Authors:** Hyung‐Jin Kim, Wa Cao, Gi‐Su Oh, SeungHoon Lee, AiHua Shen, Dipendra Khadka, Su‐Bin Lee, Subham Sharma, Seon Young Kim, Seong‐Kyu Choe, Tae Hwan Kwak, Jin‐Man Kim, Raekil Park, Hong‐Seob So

**Affiliations:** ^1^ Center for Metabolic Function Regulation (CMFR) and Department of Microbiology Wonkwang University School of Medicine Jeonbuk Korea; ^2^ NADIANBIO Ltd, Business Incubation Center Iksan Korea; ^3^ Department of Pathology and Infection Signaling Network Research Center Chungnam National University School of Medicine Daejeon Korea; ^4^ Department of Biomedical Science & Engineering, Institute of Integrated Technology Gwangju Institute of Science and Technology Gwangju Korea

**Keywords:** age‐related hearing loss, inflammation, NAD^+^, NQO1, PARPs, SIRTs

## Abstract

Age‐related hearing loss (ARHL) is a major neurodegenerative disorder and the leading cause of communication deficit in the elderly population, which remains largely untreated. The development of ARHL is a multifactorial event that includes both intrinsic and extrinsic factors. Recent studies suggest that NAD^+^/NADH ratio may play a critical role in cellular senescence by regulating sirtuins, PARP‐1, and PGC‐1α. Nonetheless, the beneficial effect of direct modulation of cellular NAD^+^ levels on aging and age‐related diseases has not been studied, and the underlying mechanisms remain obscure. Herein, we investigated the effect of β‐lapachone (β‐lap), a known plant‐derived metabolite that modulates cellular NAD^+^ by conversion of NADH to NAD^+^ via the enzymatic action of NADH: quinone oxidoreductase 1 (NQO1) on ARHL in C57BL/6 mice. We elucidated that the reduction of cellular NAD^+^ during the aging process was an important contributor for ARHL; it facilitated oxidative stress and pro‐inflammatory responses in the cochlear tissue through regulating sirtuins that alter various signaling pathways, such as NF‐κB, p53, and IDH2. However, augmentation of NAD^+^ by β‐lap effectively prevented ARHL and accompanying deleterious effects through reducing inflammation and oxidative stress, sustaining mitochondrial function, and promoting mitochondrial biogenesis in rodents. These results suggest that direct regulation of cellular NAD^+^ levels by pharmacological agents may be a tangible therapeutic option for treating various age‐related diseases, including ARHL.

## 1 INTRODUCTION

Age‐related hearing loss (ARHL) or presbycusis is the most common cause of hearing loss and sensory disability, characterized by gradual deterioration of auditory sensitivity at all frequencies, with increasing age (Revuelta et al., 2017). ARHL has emerged as a major social and public health problem, affecting around 40% of people over 65 years of age; the percentage continues to increase with age, peaking in 60%–80% of people, older than 85 years (Tu & Friedman, 2018). Although the use of hearing aids or cochlear implants improves related symptoms considerably, ARHL still remains largely untreated (Choi et al., 2016). Despite the fact that the mechanism of ARHL has remained elusive, multiple studies have demonstrated that age‐dependent alterative oxidative stress, reactive oxygen species (ROS) metabolism, up‐regulation of inflammatory responses, and mitochondrial dysfunction in parallel with cellular signaling and gene expression are implicated in this process (Fujimoto & Yamasoba, 2014; Watson, Ding, Zhu, & Frisina, 2017). Particularly, structural changes and degeneration of inner ear cells, such as sensory hair cells, spiral ganglion neurons, and stria vascularis, are characteristics of aged mammals (Revuelta et al., 2017). In addition, calorie restriction (CR) prolongs the lifespan of most mammals and delays the onset of numerous age‐associated diseases, including cancer, atherosclerosis, diabetes, nephropathy, and metabolic syndrome phenotypes (Colman et al., 2014). calorie restriction has been shown to moderately retard the progression of ARHL, lessen age‐related cochlear pathology in rats (Seidman, 2000), and improve the auditory brainstem response (ABR) of rhesus monkeys (Fowler et al., 2010). However, the molecular mechanisms underlying the beneficial effects of CR on ARHL remain incompletely understood. Metabolic alterations by stimulating metabolic regulator proteins such as SIRT1, AMP‐activated protein kinase (AMPK), and PPARγ coactivator‐1α (PGC‐1α), which occur as part of the adaptation to CR, potentially underlie the efficacy of CR (Fontana, Nehme, & Demaria, 2018). These observations suggest that alterations of mitochondrial energy metabolism play a critical role in CR response. In fact, CR has been shown to protect mitochondrial function from age‐dependent decline, reduce mtDNA damage, reverse many changes in mitochondrial gene expression, and cause metabolic changes to increase energy metabolism, delaying aging in mammals (Fontana et al., 2018).

NAD^+^ and NADH are crucial mediators of energy metabolism and cellular homeostasis, as they act as cofactors for NAD^+^‐dependent enzymes, including sirtuins (SIRTs), histones, and poly (ADP‐ribose) polymerases (PARPs) (Kim, Oh, Choe, et al., 2014). Notably, cytosolic‐free NAD^+^ levels decrease under various pathological conditions, including diabetes, aging, hypertension, and arterial restenosis (Ido, 2007; Massudi et al., 2012). There is strong evidence to support a role for SIRT1 in the process of aging and cell death, through deacetylation of targets such as NF‐κB and p53. These molecules play important roles in pro‐inflammatory responses, DNA damage, and apoptosis (Han et al., 2016), which may mediate the effects of CR in mammals (Yu, Qin, Chen, Fu, & Wang, 2018). In addition, it has been proven that SIRT3 plays key roles in mitochondrial functions through deacetylation of mitochondrial proteins, such as acetyl‐CoA synthase 2 (AceCS2), glutamate dehydrogenase (GDH), and NADH dehydrogenase [ubiquinone] 1 alpha subcomplex subunit nine (NDUFA9) (Ahn et al., 2008). A recent study shows that reduction of SIRT3 expression results in oxidative stress and mitochondrial dysfunction due to ROS production in ARHL mice (Kwon, Park, Choi, Gurunathan, & Kim, 2015). Moreover, SIRT1 activation by pharmacological agents such as resveratrol, increases mitochondrial biogenesis through SIRT1‐dependent deacetylation of the transcriptional coactivator, PGC‐1α (Desquiret‐Dumas et al., 2013). Therefore, we hypothesize that long‐term induction of high cellular NAD^+^ levels may mimic the protective effects of CR against ARHL. NQO1, one of the cytosolic antioxidant flavoproteins, catalyzes the reduction of extremely reactive quinone metabolites, using NADH as an electron donor (Ying, 2008). β‐Lap, a quinone‐containing natural compound (3,4‐dihydro‐2,2‐dimethyl‐2*H‐*naphtho [1,2‐*b*] pyran‐5–6‐dione), has recently been identified as a potent substrate of NQO1, which promotes oxidation of NADH, thereby increasing the cellular NAD^+^ levels (Kim, Oh, Shen, et al., 2014).

In this study, we elucidated the role of NAD^+^ metabolism in mice with ARHL and the effect of β‐lap on ARHL by modulating intracellular NAD^+^ levels and NAD^+^‐dependent enzymatic pathways, implicating several vital mediators.

## 2 RESULTS

### 2.1 β‐Lap ameliorates the hearing loss of aged mice and protects against apoptosis in the cochlear tissue

**Figure 1:**
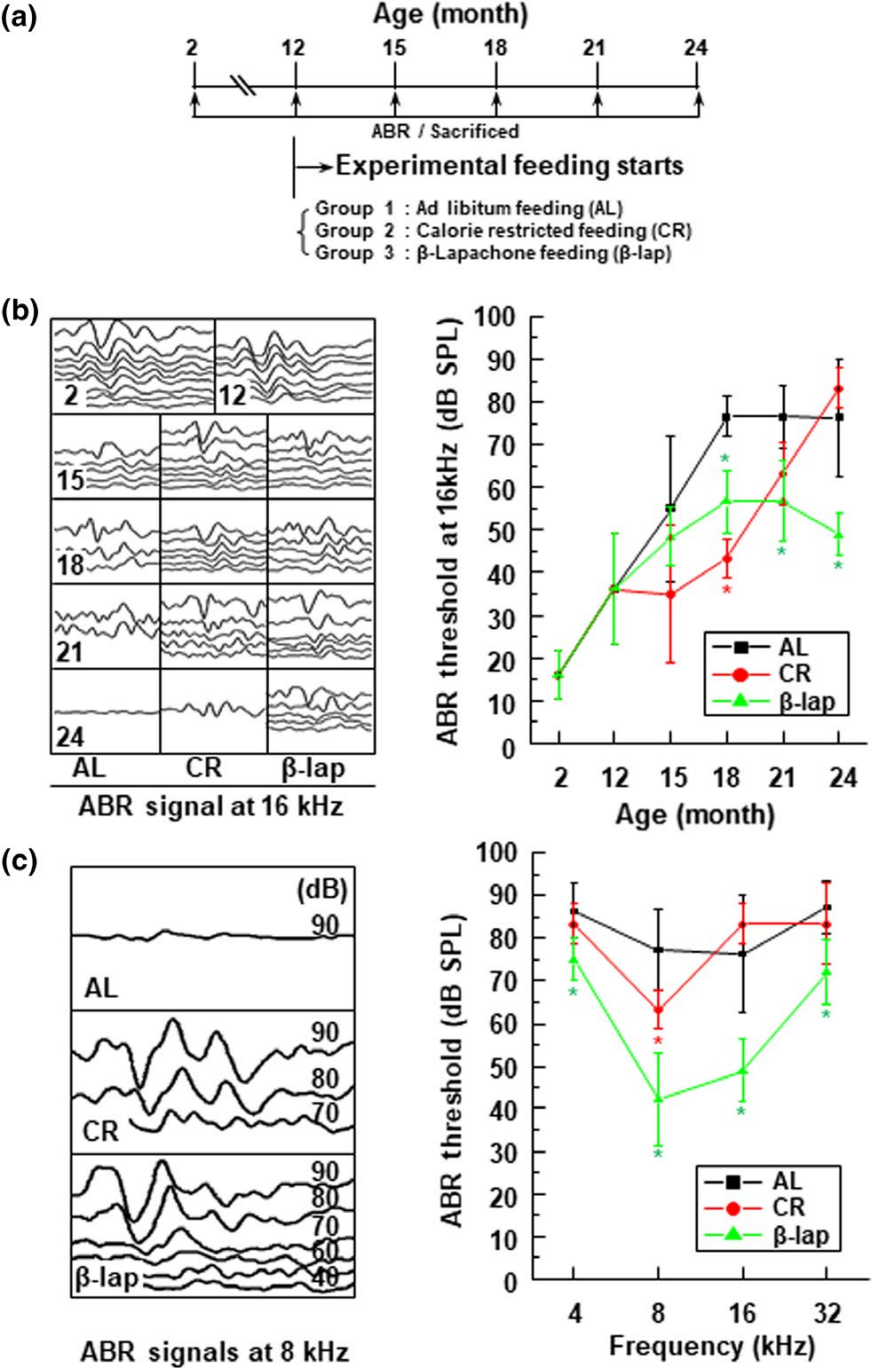
β‐Lapachone (β‐lap) improved age‐related hearing loss in a mouse model. (a) Schematic illustration of experimental schedules and groups (*n* = 13). (b) Typical changes in auditory brainstem response (ABR) signals over months, and ABR threshold shifts at 16 kHz. (c) Comparison of ABR signals under each feeding condition at 24 months, and ABR threshold shifts at different frequencies. Data are mean ± *SD*. *p < .05 versus ad libitum (AL) group for each indicated month

A schematic view of the experimental feeding design is shown in Figure 1a. To investigate the effects of β‐lap and CR on age‐related hearing loss, hearing auditory brainstem response (ABR) was performed every 3 months, from 12 to 24 months, in the experimental groups of C57BL/6J mice (Figure 1b). We observed that aging resulted in increased ABR hearing thresholds at the middle (16 kHz) frequency in mice belonging to the AL group. The AL control mice exhibited normal hearing at 2 months, gradual and significant hearing loss from 12 months, and reached the comprehensive deafness level at 24 months (Figure 1b). In contrast, mice belonging to the CR group displayed nearly normal hearing function till 15 months, after which, they displayed gradual hearing loss, and eventually reached almost deafness level at 24 months (Figure 1b). Surprisingly, the ABR thresholds of the β‐lap group gradually increased up to 15 months of age and were subsequently sustained at mean levels of 40–60 Db SPL (Figure 1b). Furthermore, obvious shifts in ABR threshold were detected in both AL and CR groups at 24 months of age, at all frequencies examined (4, 8, 16, and 32 kHz), but were strongly controlled in the β‐lap group (Figure 1c). To validate the ABR test results, we examined whether the observed loss of hearing function was associated with apoptosis in the cochlear tissues. TUNEL analysis revealed that the total number of TUNEL‐positive nuclei, showing apoptosis in the stria vascularis, spiral ligament, spiral ganglion neurons, and organ of Corti of both AL and CR groups, was clearly increased at 24 months. However, TUNEL‐positive nuclei were almost undetectable in the corresponding tissues of the 24‐month mice of the β‐lap group (Figure S1). These data indicate that apoptosis is closely related to the onset of ARHL in aged AL and CR mice. Moreover, β‐lap exerts a protective effect on hearing function and attenuates the hearing loss of aged mice by inhibiting apoptosis of cochlear tissues.

### 2.2 β‐Lap treatment sustains normal mitochondrial structure and stimulates mitochondrial biogenesis

**Figure 2:**
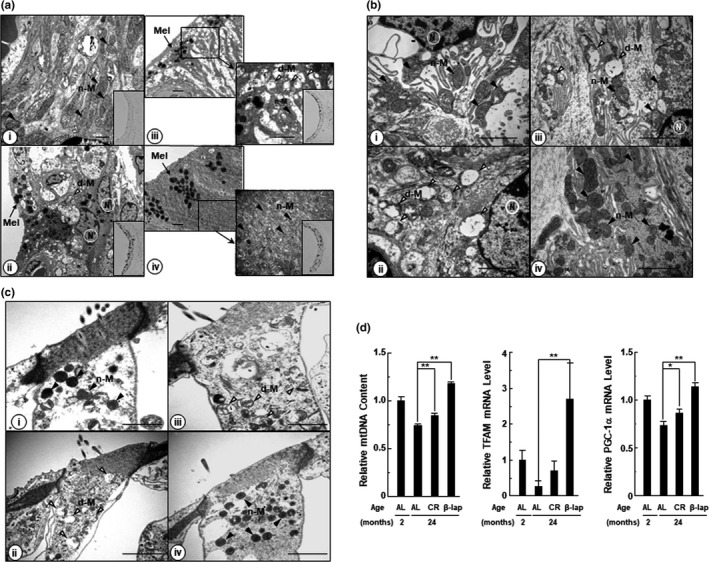
β‐Lap maintained mitochondrial structure and stimulated mitochondrial biogenesis. Transmission electron microscope (TEM) images in the stria vascularis (a), outer hair cells (b), and spiral ligament (c). i: Young mice (2 months old); ii: ad libitum‐fed (AL) aged mice (24 months old); iii: calorie‐restricted (CR) mice (24 months old); iv: β‐lap‐treated mice (24 months old). d‐M: damaged mitochondria (open arrowheads), n‐M: normal mitochondria (closed arrowheads), mel: melanin (black arrow), and N: nucleus. Scale bar: 2 μm. (d) Levels of mtDNA content and mitochondrial biogenesis‐related gene. Quantitative RT–PCR was performed as described under the "EXPERIMENTAL PROCEDURES." MtDNA content is calculated as the mtDNA/ nDNA ratio. Data are mean ± *SD* (*N* = 5). *p < .05, **p < .01 versus 24‐month‐old ad libitum‐fed mice (AL group)

Mitochondrial dysfunction is thought to play an important role in aging and age‐related diseases, including ARHL (Fujimoto & Yamasoba, 2014). Therefore, we examined the ultrastructural changes of the mitochondria in the cochlear tissues of 2‐month‐old control mice, and 24‐month‐old mice, of the three experimental groups. The mitochondria of the cochlear tissues, including stria vascularis (Figure 2a), spiral ligament (Figure 2b), and outer hair cells of Corti (Figure 2c) in 2‐month‐old control mice, exhibited normal quantity and morphology (well‐organized cristae and matrix structure). In contrast, in the corresponding cochlear tissues of the 24‐month‐old AL and CR mice, mitochondrial numbers decreased and most of the mitochondria appeared to be swollen, distorted, and deficient in cristae and matrix (Figure 2a‐c). Notably, the β‐lap‐treated mice showed an increased number of mitochondria and normal morphology in all the examined cochlear tissues (Figure 2a‐c). Strial melanin is produced by the strial intermediate cells, is exported to the melanosome in the intrastrial space, and increases with age (Ohlemiller, Rice, Lett, & Gagnon, 2009). Likewise, in our study, we observed electron‐dense spherical granules in the stria vascularis of the 24‐month‐old mice, belonging to the three experimental groups (Figure 2ai‐ii), which were confirmed as melanin, by Fontan–Masson melanin staining (small box in Figure 2aiii‐iv). However, melanin was not detected in the 2‐month‐old control mice (Figure 2ai). Interestingly, the melanin content of stria vascularis was similar in the 24‐month‐old mice of all the three groups. However, unlike mice belonging to the other two groups, which showed irregular and dispersed melanosome pattern, the β‐lap‐treated mice exhibited a regular arrangement of melanosomes. This difference may be attributed to the varying degrees of structural changes, depending on the extent of tissue damage in each group.

Mitochondrial biogenesis, a process by which cells increase mitochondrial mass, is activated by numerous signals in response to various stimuli (Scarpulla, 2008). It involves the synthesis, import, and integration of proteins and lipids to the existing mitochondrial system, as well as replication of mtDNA. Peroxisome proliferator‐activated receptor gamma coactivator 1‐alpha (PGC‐1α), the main regulator of mitochondrial biogenesis, co‐activates many transcription factors, including nuclear respiratory factor (NRF‐1 and NRF‐2). The NRFs, in turn, stimulate the mitochondrial transcription factor A (TFAM), which is a mtDNA‐binding protein and plays an essential role in genome maintenance (Lin, Handschin, & Spiegelman, 2005). Up‐regulated TFAM transcription is directly related to amplified mtDNA replication and transcription (Scarpulla, 2008). Therefore, we next examined the levels of mtDNA, PGC‐1α and TFAM. As shown in Figure 2d, the expression of mtDNA, TFAM, and PGC‐1α in the 24‐month‐old AL mice was significantly reduced as compared to the 2‐month‐old AL mice, indicating that mitochondrial biogenesis decreased with age. However, CR and β‐lap treatment elevated the levels of mtDNA, TFAM, and PGC‐1α in the 24‐month‐old mice belonging to these groups, compared to the 24‐month‐old mice of the AL group. Moreover, the effect was noticeably pronounced in the β‐lap group. Together, these results indicate that β‐lap contributes to the maintenance of normal mitochondrial structure and mitochondrial biogenesis.

### 2.3 β‐Lap suppresses ROS production and pro‐inflammatory responses in aged mice

Aging is characterized by increased oxidative stress and inflammation, and has been reported to have a relationship with ARHL and chronic inflammatory diseases, such as diabetes and cardiovascular disease (Watson et al., 2017). Additionally, previous studies have demonstrated that increased ROS production and pro‐inflammatory cytokines, such as TNF‐α, IL‐1β, and IL‐6, play critical roles in cochlear tissue damage, in noise‐ or cisplatin‐induced hearing loss (Fujioka et al., 2006; So et al., 2007). We investigated whether age‐dependent changes in ROS production and pro‐inflammatory responses in control and experimental mice were influenced by the co‐administration of β‐lap. Immunohistochemical staining of cochleae demonstrated that TNF‐α was prominently accumulated in the entire cochlear tissue, throughout the spiral ligament, spiral limbus, spiral ganglionic neurons, as well as sensory hair cells in the organ of Corti in mice belonging to the AL group since the age of 15 months, whereas these changes were almost undetectable in mice belonging to the CR group up to 15 months of age. Thereafter, a gradual and ubiquitous increase in TNF‐α expression was evident with an increase in age, in the entire cochlear region of the mice belonging to the CR group (Table S1 and Figure S2). Surprisingly, slight TNF‐α expression was detected only in the cochlear region of 24‐month‐old mice belonging to the β‐lap group. We examined the presence of a well‐known marker of oxidative damage in cellular DNA, 8‐hydroxy‐2'‐deoxyguanosine (8OHdG), which is an indicator of excessive ROS production (Kasai, 1997). It was found to be increased in the cochlear tissues of 24‐month‐old mice, belonging to the AL and CR groups, but was attenuated in mice belonging to the β‐lap group (Figure S3). In addition, IL‐1β, IL‐6, NF‐κB, as well as ROS generation‐related molecules, such as NOX3, HMGB‐1, AGEs, and RAGE in the cochlear regions of the mice belonging to the AL and CR groups, exhibited expression patterns similar to those of TNF‐α, with increasing age (Table S1). However, β‐lap notably delayed and decreased the expression of these molecules, which were detected in small amounts only in the cochlear tissues of the 24‐month‐old mice (Table S1). Consistent with the histological observation results, mRNA and protein expression of TNF‐α, IL‐1β, and IL‐6 were significantly increased in total cochlear tissues of the 24‐month‐old mice belonging to the AL and CR groups, compared to those of the 2‐month‐old control mice (Figure S4). However, β‐lap almost completely blocked the increase in both, mRNA and protein expression, of these inflammatory molecules. These results indicate that β‐lap mitigated the accumulation of multiple pro‐inflammatory cytokines as well as ROS production, and protected against oxidative damage in the cochlear tissues of aged mice.

### 2.4 β‐Lap increases the intracellular NAD^+^ levels, attenuates PARP‐1 hyperactivation, and restores SIRT1 activity

**Figure 3:**
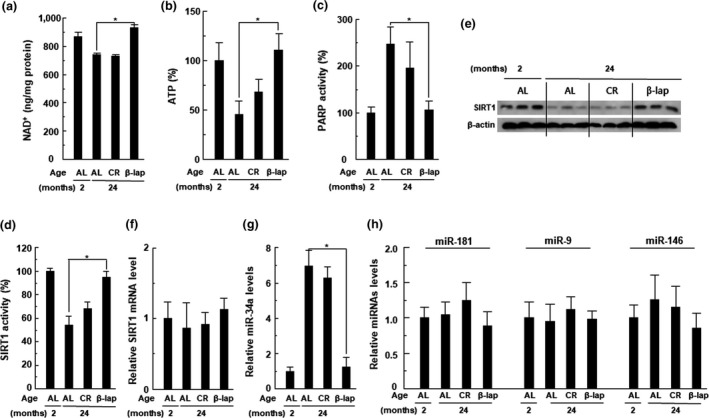
β‐Lap restores the NAD^+^ and SIRT1 activity in cochlear tissues. (a) NAD^+^ was extracted from cochlear tissues, and changes in NAD^+^ levels were measured using a fluorescent NAD^+^ detection kit. (b) PARP activity was assayed using the PARP assay kit. (c) Intracellular ATP levels in the cochlear tissue were measured using ATP assay kit, on a luminometer. (d) SIRT1 activity was measured using SIRT1 assay kit. (e) Level of SIRT1 protein was analyzed by Western blotting. (f) Level of SIRT1 mRNA was measured by qRT–PCR. (g) Level of miR‐34a was measured by qRT–PCR. (h) Levels of miR‐181, miR‐9, and miR‐146 were analyzed by qRT–PCR. Each value represents the mean ± *SD* (*n* = 5). *p < .05 versus 24‐month‐old ad libitum‐fed mice (AL group)

PARP1 hyperactivation by DNA damage is considered to result in depletion of cellular NAD^+^ and ATP levels (Bai et al., 2011). Many studies have revealed that the beneficial properties of β‐lap on multiple metabolic syndromes and cisplatin‐mediated ototoxicity/nephrotoxicity were mediated by increased intracellular NAD^+^ levels and SIRT1 activation by inhibiting PARP hyperactivation (Kim, Oh, Shen, et al., 2014; Wu et al., 2016). Because PARP1 and SIRTs compete with the same cellular NAD^+^ pool, PARP1 activation has a significant effect on SIRTs activity by decreasing NAD^+^ bioavailability (Bai et al., 2011). Recently, the intracellular NAD^+^ level has been reported to be decreased in various organs, including liver, heart, kidney, and lung, in aged animals and human skin tissue. This has been attributed to PARP1 hyperactivation induced by the accumulation of age‐related oxidative stress and consequent DNA damage (Massudi et al., 2012). Indeed, in this study, we observed a significant decrease in NAD^+^ and ATP levels in total cochlear tissues of the 24‐month‐old mice belonging to the AL and CR groups compared to the 2‐month‐old control mice; however, this reduction was restored by β‐lap co‐administration (Figure 3a,c). Consistent with this finding, we observed that activation of PARP in aged mice was significantly inhibited by β‐lap (Figure 3b). SIRT1, an NAD^+^‐dependent deacetylase, has been implicated in age‐related diseases (Han et al., 2016). Therefore, we evaluated whether β‐lap affects SIRT1 activity and expression in ARHL mice. Our results show that SIRT1 protein expression and activity were significantly reduced in total cochlear tissues of the 24‐month‐old mice, belonging to the AL and CR groups, compared to the 2‐month‐old control mice, albeit, both were restored by β‐lap (Figure 3d‐e). Immunohistochemical staining to examine the expression of SIRT1 in the cochleae showed similar results (Figure S5). However, SIRT1 mRNA levels were not different in the three experimental groups (Figure 3f). We therefore investigated whether altered expression of miR‐34a, miR‐181, miR‐9, and miR‐146, which are known to lower the level of SIRT1 protein by translational inhibition (Nogueiras et al., 2012), is associated with a decrease in SIRT1 protein levels. Of note, the expression level of miR‐34a was significantly increased in the 24‐month‐old mice of the AL and CR groups; however, this increase was completely attenuated by β‐lap co‐administration, to the level observed in the 2‐month‐old mice, belonging to the control group (Figure 3g). However, the expression levels of miR‐181, miR‐9, and miR‐146 were not significantly different among the 2‐month‐old control group and the three 24‐month‐old experimental groups (Figure 3h). These data imply that miR‐34a may function as a post‐transcriptional negative modulator that can determine the level of SIRT1 protein in cochlear tissue of aged mice. Furthermore, these data also suggest that an increase in SIRT1 activity by β‐lap is due to an increase in NAD^+^ levels and SIRT1 protein expression by β‐lap.

### 2.5 β‐lap alleviates CM‐induced decrease of SIRT1 and decreases acetylated p53 and miR‐34a expression

**Figure 4:**
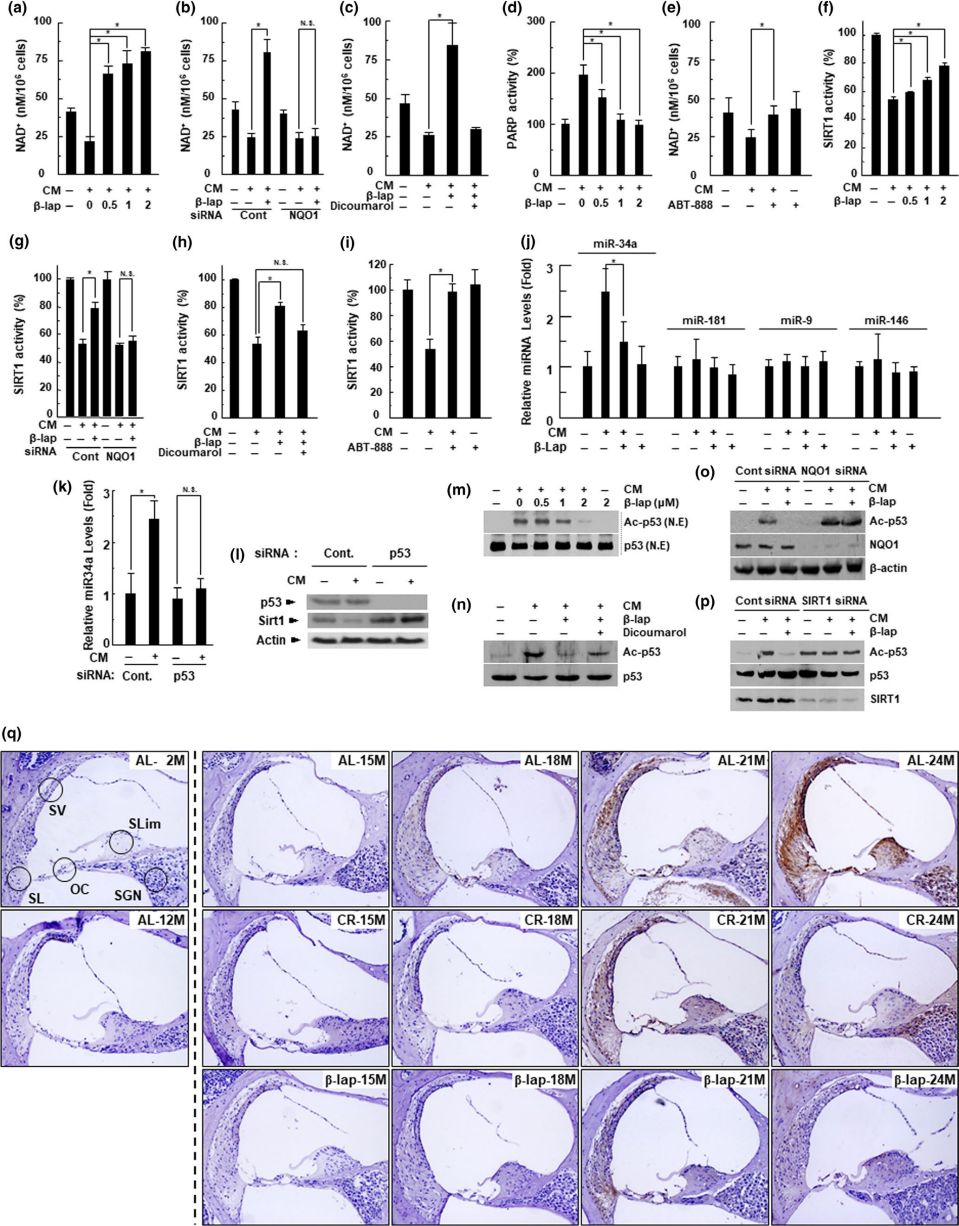
β‐Lap restores the NAD^+^ and SIRT1 activity in HEI‐OC1 cells. HEI‐OC1 auditory cells were treated with CM in the presence of indicated β‐lap dose for 24 hr (a, d, f, j). Then, we measured the changes in NAD^+^ levels using a fluorescent NAD^+^ detection kit (a), PARP activity using the PARP assay kit (d), SIRT1 activity using the SIRT1 assay kit (f), levels of miR‐34a, miR‐181, miR‐9, and miR‐146 by qRT–PCR (j). HEI‐OC1 auditory cells were pretreated with dicoumarol (NQO1 enzymatic inhibitor, 10 μM) (c, h) for 1 hr. These cells were treated with CM with or without β‐lap (1 μM) for 24 hr. Then, NAD^+^ levels (c) and SIRT1 activity (h) were measured. HEI‐OC1 auditory cells were pretreated with ABT‐888 (PARP inhibitor, 1 μM) (e, i) for 1 hr. These cells were treated with CM for 24 hr. Then, NAD^+^ levels (e) and SIRT1 activity (i) were measured. HEI‐OC1 auditory cells were transfected with siRNAs against NQO1(b, g) or p53 (k) for 36 hr, and then stimulated with CM for 24 hr in the absence or presence of β‐lap (1 μM). Then, NAD^+^ level (b), SIRT1 activity (g), and miRNA34a level (k) were measured. Each value represents the mean ± *SD* (*n* = 5). *p < .05. HEI‐OC1 auditory cells were transfected with scrambled control siRNA or siRNA against p53 for 36 hr, and then stimulated with CM for 24 hr. Then, Western blot analyses were performed using antibodies for p53, SIRT1, and β‐actin (l). HEI‐OC1 auditory cells were treated with CM in the presence of indicated β‐lap dose for 24 hr. Then, nuclear fractions were prepared and Western blot analyses were performed using antibodies for p53 and ac‐p53 (m). HEI‐OC1 auditory cells were pretreated with dicoumarol for 1 hr. These cells were treated with CM in the presence of indicated β‐lap dose for 24 hr. Then, Western blot analyses were performed using antibodies for p53 and ac‐p53 (n). HEI‐OC1 auditory cells were transfected with scrambled control siRNA or siRNA against NQO1 for 36 hr, and further stimulated with CM for 24 hr in the absence or presence of β‐lap. Then, Western blot analyses were performed using antibodies for ac‐p53, NQO1, and β‐actin (o). HEI‐OC1 auditory cells were transfected with scrambled control siRNA or siRNA against SIRT1 for 36 hr, and further stimulated with CM for 24 hr in the absence or presence of β‐lap. Then, Western blot analyses were performed using antibodies for ac‐p53, p53, and SIRT1 (p). Cochleae were removed from C57BL/6J mice that were fed with either AL, CR diet, or β‐lap‐supplemented diet. Thereafter, the cochleae were decalcified and embedded in paraffin. Next, 5‐μm‐thick sections were prepared. Then, immunohistochemical staining was performed for acetylated p53 in mouse cochleae. All procedures are described in “EXPERIMENTAL PROCEDURES” section. OC, organ of Corti; SG, spiral ganglion neuron; SL, spiral ligament; SV, stria vascularis

HEI‐OC1 cells, one of the few mouse auditory cell lines available for research purposes, were used to directly examine the effect of β‐lap on mediators involved in ARHL, such as NAD^+^, PARP, and SIRT1. In a previous study, we clearly described the role of pro‐inflammatory cytokines, including TNF‐α, IL‐1β, IL‐6, and NF‐κB, in the pathogenesis of cisplatin ototoxicity in vitro and in vivo (So et al., 2007). In this study, we demonstrated that these pro‐inflammatory cytokines in cochlear tissues increased with age. Therefore, we considered that in vitro treatment of HEI‐OC1 auditory cells with a pro‐inflammatory cytokine mixture (CM, comprising TNF‐α, IL‐1β, and IL‐6 at 20 ng/ml, each) would mimic the pathological aging process of cochlear tissues. Indeed, cellular NAD^+^ level was significantly decreased by CM, but β‐lap significantly restored NAD^+^ level in a dose‐dependent manner (Figure 4a). However, this restoring effect of β‐lap was markedly blocked by transfection with NQO1‐specific siRNA, or co‐administration with dicoumarol, a NQO1 enzymatic inhibitor (Figure 4b,c). In addition, the PARP activity in HEI‐OC1 cells was clearly increased by CM; however, this increase was significantly inhibited by β‐lap in a dose‐dependent manner (Figure 4d). The decrease in intracellular NAD^+^ level induced by CM was significantly alleviated by the PARP inhibitor ABT‐888 (Figure 4e). Next, we examined whether SIRT1 activity was altered by CM treatment, and whether β‐lap or PARP1 inhibitor was able to modulate such changes induced by CM. As expected, SIRT1 activity, after CM exposure, was significantly reduced, while β‐lap or ABT‐888 significantly alleviated this reduction (Figure 4f and 4i). The positive effect of β‐lap on SIRT1 activity was abolished by the knockdown of NQO1, using NQO1‐specific siRNA or by dicoumarol (Figure 4g,h). Collectively, these data indicate that β‐lap acts as a substrate for NQO1, thereby increasing cellular NAD^+^ levels and SIRT1 activity, in an NQO1‐dependent manner. On the other hand, similar to the in vivo results, miR‐34a expression was significantly increased by CM in HEI‐OC1 cells, and this increase was completely inhibited by β‐lap. However, the levels of miR‐181, miR‐9, and miR‐146 were not affected by CM or β‐lap (Figure 4j). Activation mechanisms of p53, such as p53 acetylation, are considered key mediators and upstream regulators of miR‐34a, and play an important role in aging‐related diseases, by modulating p53‐dependent activation of apoptosis and senescence (Yamakuchi & Lowenstein, 2009). In our study, increased CM‐induced miR‐34a and decreased SIRT1 expression were significantly blocked by p53 knockdown (Figure 4k,l). In addition, CM treatment of HEI‐OC1 cells significantly increased p53 acetylation, without causing a change in the total p53 protein level; however, β‐lap significantly inhibited p53 acetylation (Figure 4m). Immunohistochemical staining also showed that β‐lap apparently blocked acetylated p53 in the cochlear tissues of 24‐month‐old mice (Figure 4q). However, this effect of β‐lap, in vitro*,* was significantly attenuated by dicoumarol as well as knockdown of NQO1 or SIRT1, using siRNA (Figure 4n‐p).

### 2.6 **β‐Lap prevents NF‐κB activation by inhibiting I**κB phosphorylation and NF‐κB p65 acetylation

**Figure 5:**
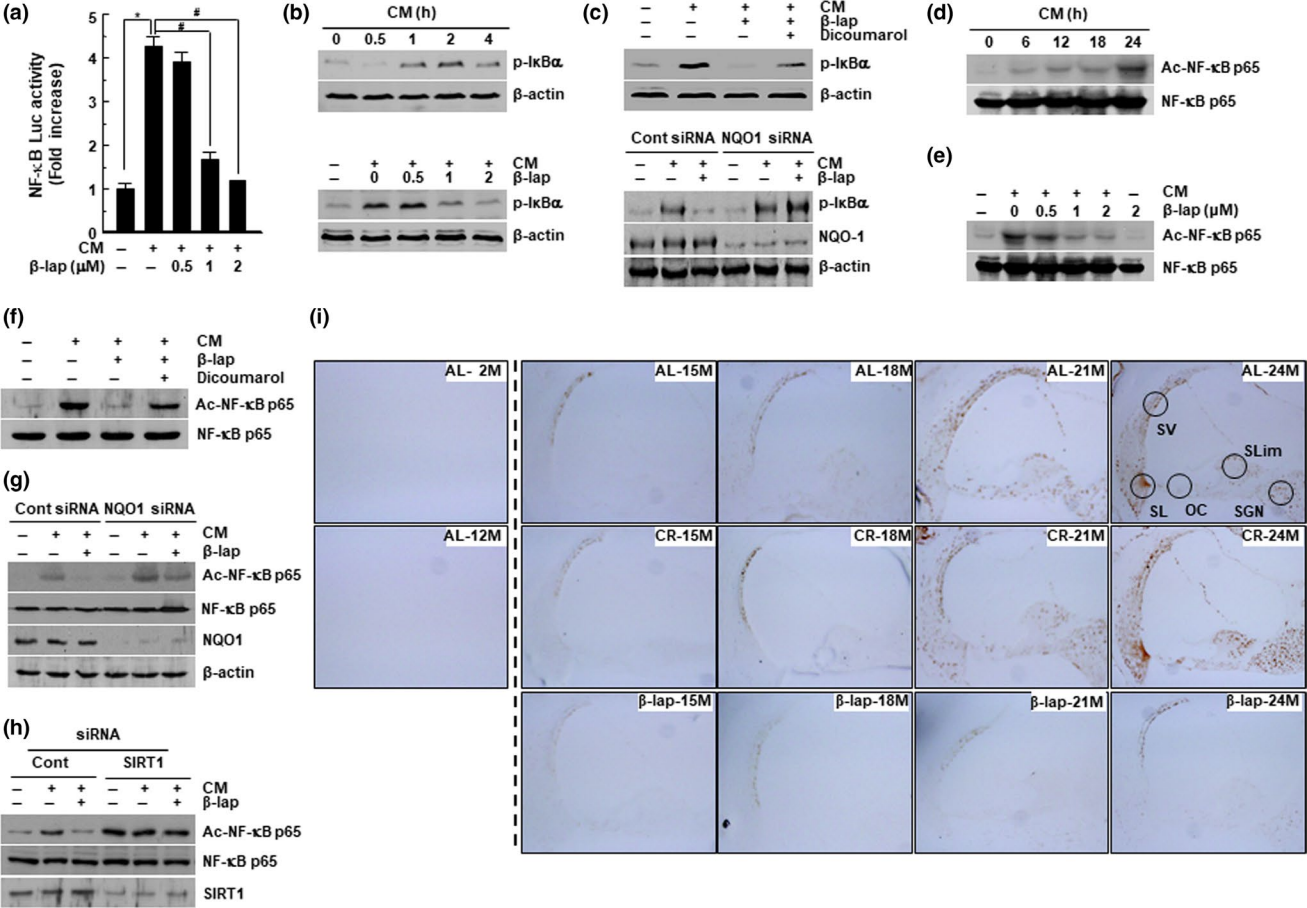
β‐Lap inhibits NF‐κB activation in HEI‐OC1 cells. (a) HEI‐OC1 cells were treated with cytokine mixture (CM) in the presence or absence of β‐lap, following which transcriptional activities of NF‐κB were analyzed by luciferase reporter assay. Results are represented as mean ± *SD* (*N* = 5). *p < .05 versus control cells, #*p* < .05 versus CM alone cells. (b‐h) HEI‐OC1 cells were treated with CM, β‐lap, dicoumarol, siRNAs for NQO1 and SIRT1, at the indicated conditions. After preparation of whole‐cell lysates, Western blot analyses were performed using antibodies for p‐IκBα, NQO1, ac‐NF‐κB p65, NF‐κB p65, SIRT1, and β‐actin. (i) Immunohistochemical staining for acetylated NF‐κB p65 in mouse cochleae. Cochleae were removed from C57BL/6J mice fed on either AL diet, CR diet, or a β‐lap‐supplemented diet. Thereafter, the cochleae were decalcified and embedded in paraffin. Next, 5‐μm‐thick sections were prepared. All immunohistochemical staining procedures are described in “EXPERIMENTAL PROCEDURES” section

NF‐κB is an important factor in the inflammatory responses, and activation of the NF‐κB pathway leads to the transcription of numerous genes, including pro‐inflammatory cytokines, which are closely associated with age‐related diseases (Kubben & Misteli, 2017). As β‐lap attenuates inflammatory responses in aged mice, we next examined the effect of β‐lap on NF‐κB activation. NF‐κB activity was apparently increased by CM treatment in HEI‐OC1 cells; however, this increase was significantly reduced by β‐lap in a dose‐dependent manner (Figure 5a). Furthermore, we observed that β‐lap inhibited CM‐induced phosphorylation of IκBα and NF‐κB p65 acetylation in a dose‐dependent manner; however, this effect of β‐lap was significantly blocked by knockdown of NQO1 using siRNA or dicoumarol in HEI‐OC1 cells (Figure 5b‐g). In particular, when SIRT1 protein expression was impeded by SIRT1‐specific siRNA, the deacetylation effect of β‐lap on acetylated NF‐κB p65 was completely blocked (Figure 5h). Additionally, immunohistochemical staining demonstrated that β‐lap significantly inhibited the acetylation of NF‐κB p65 in the cochlear tissues of 24‐month‐old mice compared to the mice of the AL and CR groups (Figure 5i). Taken together, these results suggest that β‐lap inhibits CM‐induced NF‐κB activity by inhibiting IκBa phosphorylation and NF‐κB p65 acetylation in an NQO1‐dependent manner and an NQO1/SIRT1‐dependent manner, respectively.

### 2.7 β‐lap restores mitochondrial function by regulating the CM‐induced down‐regulated activity of SIRT3 and ETC complex I by modulating the acetylation of p53 and IDH2

**Figure 6:**
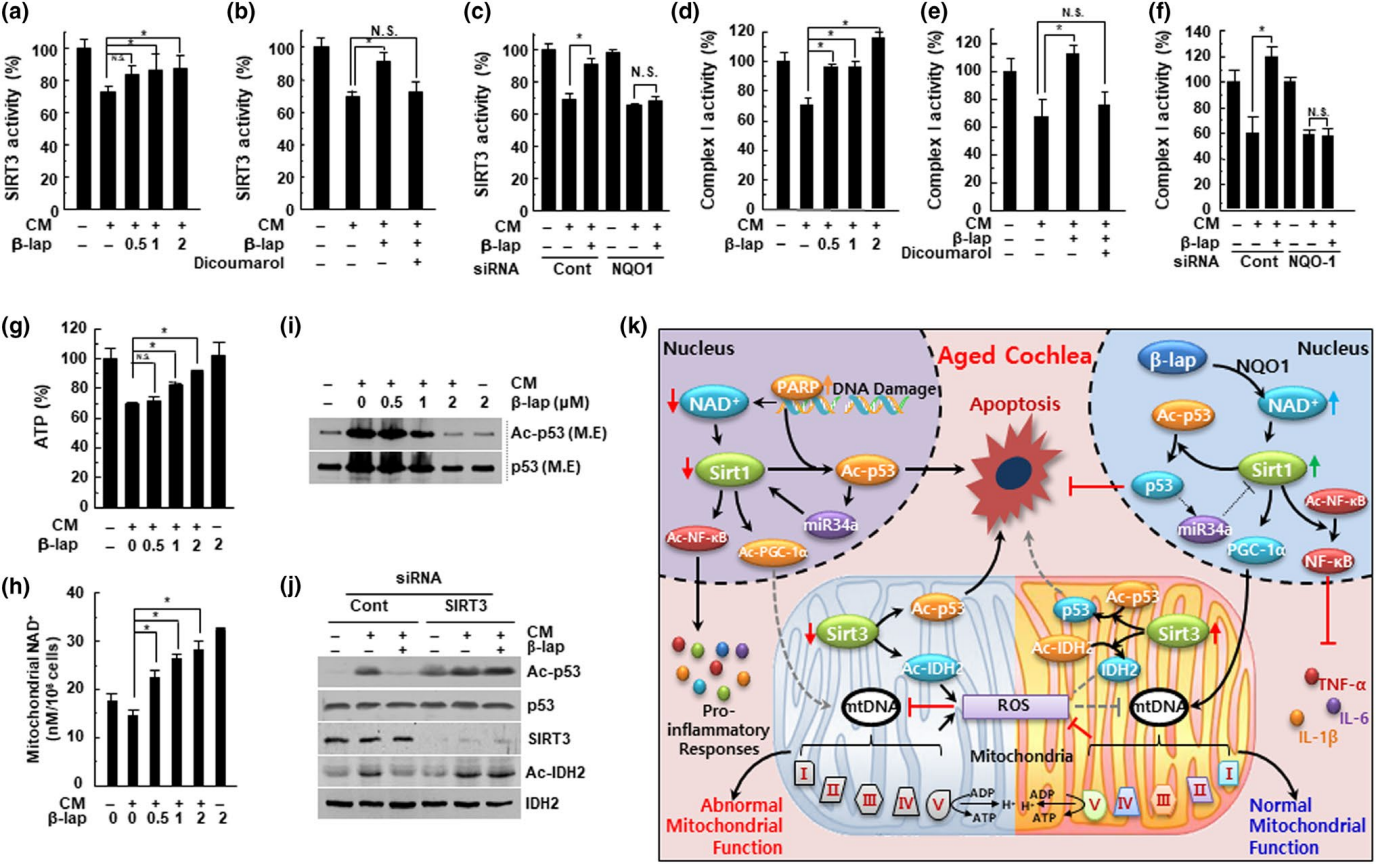
β‐Lap attenuated the CM‐mediated reduction of SIRT3 and mitochondrial complex I activities. HEI‐OC1 cells were pretreated with β‐lap, dicoumarol, siRNA for NQO‐1, or SIRT3 and thereafter stimulated with CM for 24 hr. (a‐c) SIRT3 activity was measured using a fluorescent SIRT3 assay kit. (d‐f) Mitochondrial electron transport chain complex I activity was measured using the spectrophotometric method. (g) Cellular ATP level was measured using a luminescent ATP assay kit. *p < .05 versus CM‐treated cells, N.S: not significant, Data are represented as mean ± *SD* (*N* = 5). (h) Mitochondrial NAD^+^ levels were measured using a fluorescent NAD^+^ detection kit. (i‐j) p53 acetylation status was assessed from mitochondrial protein extracts, by Western blotting. Endogenous acetylated isocitrate dehydrogenase 2 (IDH2) was detected by immunoprecipitation with anti‐IDH2 antibody, followed by Western blotting with anti‐acetyl‐lysine antibody (j, bottom). M.E: mitochondrial extract. (k) Proposed mechanism for β‐lap in the prevention of ARHL. Age‐associated reduction in the cellular NAD^+^ content decreases SIRT1 and SIRT3 activity, thereby increasing inflammation and ROS generation. This, in turn, leads to mitochondrial dysfunction and auditory cell death. However, NAD^+^ augmentation by β‐lap effectively prevents ARHL through the regulation of accompanying deleterious effects in mammals

Recently, it has been reported that SIRT3 is involved in the suppression of ROS, activation of mitochondrial function, and mitochondrial biogenesis, through deacetylation of its targets such as AceCS2, NDUFA9, and isocitrate dehydrogenase 2 (IDH2) (Ahn et al., 2008). SIRT3 deficiency promotes mitochondrial dysfunction (Porter, Urciuoli, Brookes, & Nadtochiy, 2014). In addition, it is generally known that mitochondrial ETC complex I (NADH: ubiquinone oxidoreductase) is essential for oxidative phosphorylation in mammalian mitochondria (Slater, 2003). Therefore, to further investigate the protective effects of β‐lap on mitochondrial function, we examined the SIRT3 and complex I activity localized in the mitochondria. We found that SIRT3 and complex I activity as well as ATP and mitochondrial NAD^+^ levels were significantly decreased when treated with CM. Furthermore, β‐lap abolished these inhibitory effects induced by CM in a dose‐dependent manner (Figure 6a,d,g,h). However, the preventive effects of β‐lap on the CM‐mediated decrease in SIRT3 and complex I activity were completely blocked by the NQO1 enzymatic inhibitor, dicoumarol (Figure 6b,e, respectively), and NQO1‐specific siRNAs (Figure 6c,f). In addition, we found that β‐lap attenuated the acetylation of mitochondrial p53 and IDH2 induced by CM (Figure 6i,j). Nonetheless, this decrease was completely restored by SIRT3 knockdown (Figure 6j).

## 3 DISCUSSION

In this study, we demonstrated apoptosis, mitochondrial dysfunction, ROS production, and accumulation of inflammatory cytokines, in 24‐month‐old mice that were given AL and CR diet. We also revealed that these age‐related pathophysiologic changes are mediated by both decreased intracellular NAD^+^ level due to PARP‐1 hyperactivation and down‐regulated SIRT1 expression via the p53‐miR‐34a pathway, which leads to apparent decrease in SIRT1 and SIRT3 activity. Furthermore, we demonstrated that due to decreased expression, SIRT1 and SIRT3 failed to deacetylase their targets, p53, NF‐κB, complex I, and IDH2 (which are activated by acetylation), in aged mice in vivo*,* or in CM‐treated HEI‐OC1 cells, in vitro. Lessened deacetylation augmented inflammation, apoptosis, and mitochondrial dysfunction, thereby aggravating hearing impairment. We also observed that CR attenuated ARHL by 21 months of age, but did not prevent rapid decline in hearing function, thereafter. In addition, the expression of SIRT1 in the CR group remained comparable with that in the control group, from 18 months to 21 months, with remarkable hearing function, but thereafter an obvious decline was noted. Therefore, these findings suggest that the CR is effective in preventing ARHL over a limited time period, due to age‐related decrease in both NAD^+^ and SIRT1/3. However, we found that β‐lap consistently and effectively increased cellular NAD^+^ levels and restored SIRT1 and SIRT3 expression and activity, in an NQO1‐dependent manner. This SIRT activation also promoted deacetylation of p53, NF‐κB, ETC complex I, and IDH2, leading to sustained reduction of inflammation and apoptosis, sustained normal mitochondrial function, and finally beneficial protective effects against ARHL.

Many studies have reported that β‐lap induces an undesired futile redox cycle, which is dependent on NQO1, resulting in an imbalance of redox cycle and induces oxidative stress to DNA by intracellular ROS production, which sequentially leads to PARP activation in cancer cells (Reinicke et al., 2005; Morales et al. 2014). Of note, NQO1 is commonly overexpressed in most solid tumors, particularly in non‐small‐cell lung (NSCLC), prostate, pancreatic, and breast (Bentle, Reinicke, Bey, Spitz, & Boothman, 2006; Bey et al., 2007; Dong et al., 2010). However, normal cells have lower NQO1 and are more β‐lap insensitive than cancer cells (Kung, Lu, & Chau, 2014). In addition, β‐lap has beneficial effects in many in vivo disease models such as health decline in aged mice (Lee et al., 2012), hypertension (Kim et al., 2011), arterial restenosis (Kim et al., 2009), and cisplatin‐induced hearing impairment (Kim, Oh, Choe, et al., 2014; Kim, Oh, Shen, et al., 2014). Interestingly, in our previous report, we demonstrated that the beneficial effects of β‐lap were mediated by increased levels of NAD^+^ and ATP in tissues rather than hyperactivation of PARP1 by NQO1 enzyme reaction. On the contrary, we found that these effects are mediated by PARP inactivation through inhibiting intracellular ROS production (Kim, Oh, Choe, et al., 2014; Kim, Oh, Shen, et al., 2014). These studies indicate that β‐lap can have completely conflicting effects depending on the situation.

SIRT proteins use NAD^+^ as a substrate to deacetylase various targets, including histones and NF‐κB p65 and p53 (by SIRT1) in the nucleus, and p53, IDH2, and ETC complex I (by SIRT3) in the mitochondria (Caron et al., 2014; Nogueiras et al., 2012). Furthermore, expression of SIRT1 can be regulated by miRNAs; among them, miR‐34a is the first identified miRNA to bind directly to the 3'‐UTR of SIRT1, thereby inhibiting mRNA translation and regulating apoptosis and cellular senescence (Hermeking, 2010). In addition, miR‐34a is known to be the downstream target of p53 (Yamakuchi & Lowenstein, 2009); activation of transcription factor p53 (particularly its acetylated form) causes important cellular reactions, related to oxidative stress and DNA damage, inducing sequence‐specific DNA binding and subsequent recruitment of other transcription cofactors in the nucleus. These events further promote the transcription of target genes that are involved in apoptosis, ROS production, and mitochondrial dysfunction (Reed & Quelle, 2014). In this study, we showed a significant increase in miR‐34a expression, modulated by nuclear‐localized acetylated p53 in aged mice, and CM‐treated HEI‐OC1 cells. In addition, depletion of p53 by *p53*‐specific siRNA markedly restored SIRT1 expression, via decreasing miR‐34a in CM‐treated HEI‐OC1 cells. SIRT1 also regulates p53 function through deacetylation and p53 stabilization. Our results suggest a vicious feedback loop, “cause and effect,” in which p53 induces expression of miR‐34a which suppresses SIRT1, which then boosts an increase in p53 acetylation and p53 activity. However, β‐lap significantly inhibited acetylated p53, through elevation of SIRT1 and intracellular NAD^+^ levels, and further suppressed p53‐mediated apoptosis, resulting from oxidative stress and DNA damage.

NF‐κB transcription factor is considered as the master regulator of the inflammatory response, which is associated with the development and pathogenesis of various age‐related diseases, including ARHL (Kubben & Misteli, 2017; Watson et al., 2017). According to a recent report, daily fasting (a type of dietary restriction) protects the rat heart against age‐induced inflammation and fibrosis, by suppressing NF‐κB activation and oxidative damage, indicating the preventive effect of NF‐κB inhibition, on age‐related diseases (Castello et al., 2010). NF‐κB activation is attained either by an IκB‐dependent pathway, via IκB phosphorylation, and subsequent degradation, or by an IkB‐independent pathway, via post‐translational modifications of Rels, including acetylation of the NF‐κB p65 subunit (Yeung et al., 2004). Particularly, NAD^+^‐dependent SIRT1 physically interacts with nuclear translocated NF‐κB p65 and deacetylates NF‐kB p65 at Lys‐310 residue, thereby lessening the transcriptional activity of NF‐κB (Yeung et al., 2004). Our previous studies have shown that β‐lap attenuates cisplatin‐mediated hearing impairment, by down‐regulating the acetylation of NF‐κB p65, as well as inhibiting the production of pro‐inflammatory cytokines (Kim, Oh, Shen, et al., 2014). In this study, we found that increased NF‐κB p65 acetylation in CM‐treated HEI‐OC1 cells was inhibited by increased cellular NAD^+^ levels and subsequent SIRT1 activation, after β‐lap treatment. In addition, it is noteworthy that β‐lap can also prevent NF‐κB activation by inhibiting IκB phosphorylation, although the effect of β‐lap on upstream signaling of IkB phosphorylation, including IKKs, needs to be further elucidated. Consistent with our result, Tzeng et al reported that β‐lap restrained LPS‐stimulated NF‐κB activation in alveolar macrophages, by inhibiting IκB degradation (Tzeng et al., 2003).

As previously mentioned, mitochondrial dysfunction is associated with age‐related hearing loss in mice. The most widely studied mitochondrial sirtuin, SIRT3, has been shown to regulate the activity of major enzymes in the mitochondrial metabolic pathways, such as IDH2 and AceCS2 (Ahn et al., 2008). We revealed that β‐lap could alleviate the decreased activity of SIRT3 and ETC complex I, induced by CM in vitro, and parallelly decrease the acetylation of mitochondrial p53 and IDH2, through increased deacetylase activity of SIRT3.

In particular, we have shown, for the first time, the effect of SIRT3 on the deacetylation of mitochondrial p53 in CM‐induced HEI‐OC1 cell senescence. It has been previously reported that p53 can regulate SIRT1 expression through the p53‐miR‐34a‐SIRT1 pathway (Yamakuchi & Lowenstein, 2009); however, it is not yet clear whether p53 can regulate SIRT3 expression.

Previous studies have demonstrated a link between PGC‐1α and SIRT3 expression in the process of regulating mitochondrial function and biogenesis, but the underlying signaling cascades are not fully determined (Kong et al., 2010). Kong et al. reported that PGC‐1α increased SIRT3 activity, and SIRT3 knockdown attenuated the regulation of mitochondrial biogenesis by PGC‐1α. Furthermore, as both SIRT1 and SIRT3 regulate the same process, it may be speculated that sirtuins can coordinate with one another in various cellular compartments to actively control the corresponding signals. This implies a possible crosstalk between nuclear and mitochondrial sirtuins. Therefore, it is crucial to clarify the mechanistic aspects of SIRT3 gene expression and interplay between modulating factors in order to get a full picture of the role of SIRT3 in aging and age‐related diseases.

In conclusion, as shown in Figure 6j, our results demonstrate that aging‐associated reduction in the cellular NAD^+^ level may be an important contributing factor to the pro‐inflammatory responses in cochlear tissue leading to ARHL caused by damage to mitochondrial structure and function through the decline in SIRT1 and SIRT3 activity. Notably, the increase of intracellular NAD^+^ by β‐lap effectively prevents ARHL and the accompanying deleterious effects, in mice. Our study suggests that increasing NAD^+^ with pharmacological agents could be a promising and tangible therapeutic strategy for various diseases, including ARHL.

## 4 EXPERIMENTAL PROCEDURES

### 4.1 Experimental design

To examine the effect of CR on ARHL, C57BL/6 mice were randomly divided into three experimental groups. The first group of C57BL/6 mice received ad libitum regular chow (AL group). The second group of C57BL/6 mice received only 70% of the regular chow (calorie‐restricted; CR). The third group of C57BL/6 mice received ad libitum regular chow supplemented with β‐lap (β‐lap group: 0.06% of the regular chow weight). The feeding regimen began at 12 months of age and was maintained until 24 months of age. Auditory function was measured based on the auditory brainstem response (ABR) every 3 months, from 12 to 24 months of age. After the ABR test, blood was collected, after which the whole temporal bone was removed from all the animals of the three experimental groups. The body weight was determined at 24 months of age.

## CONFLICT OF INTEREST

None declared.

## AUTHORS’ CONTRIBUTION

H.K. performed and analyzed all histological data and pharmacological stimulation for NQO1 and was involved in the writing of the manuscript. W.C. was also involved in the writing of the manuscript. G.O., S.L., A.S., D.K., S.L., S.S., and S.K. analyzed all the enzymatic activity and effects of pharmacologic stimulation of NQO1 in the in vitro or in vivo aging process experiments. S.C., T.K., and R.K. performed and analyzed all biochemical assay results that were derived from the knockdown of NOQ1, SIRT1, and SIRT3. J.K. performed electron microscopic analysis of all mouse tissues. H.S. supervised the project, developed the theory, and wrote the manuscript.
