# Supplementary figures and images for: Augmentation of cellular NAD+ by NQO1 enzymatic action improves age‐related hearing impairment
Source: Aging Cell. 2019 Jul 28;18(5):e13016. doi: 10.1111/acel.13016 (PMC6718544; doi:10.1111/acel.13016)

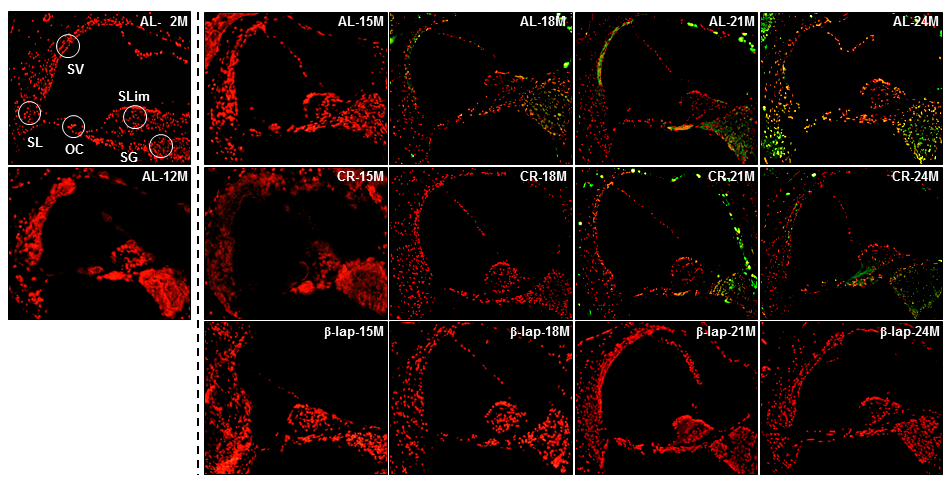

Supplement: Supplementary file 1 [file ACEL-18-e13016-s001.tif]

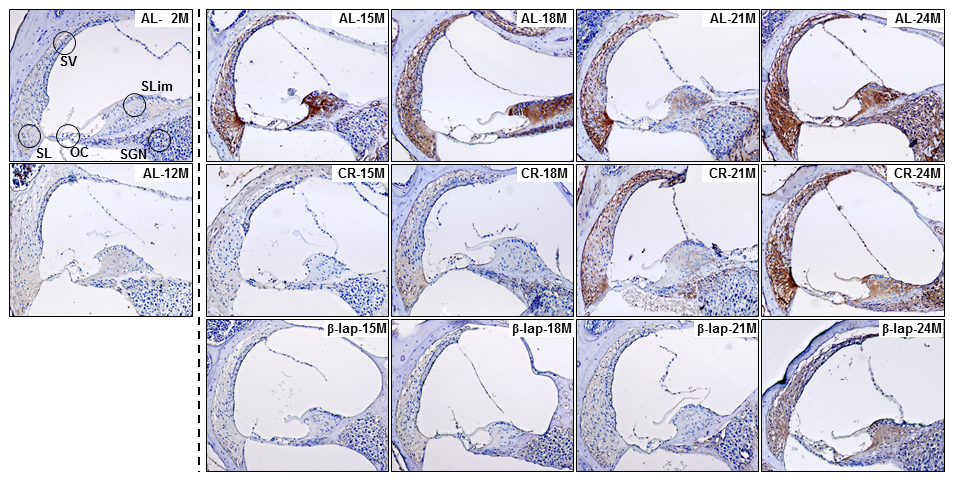

Supplement: Supplementary file 2 [file ACEL-18-e13016-s002.tif]

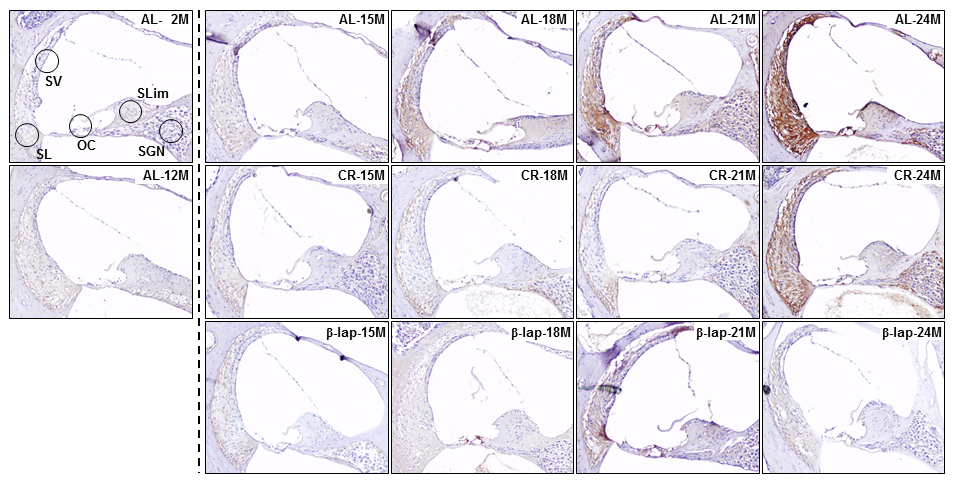

Supplement: Supplementary file 3 [file ACEL-18-e13016-s003.tif]

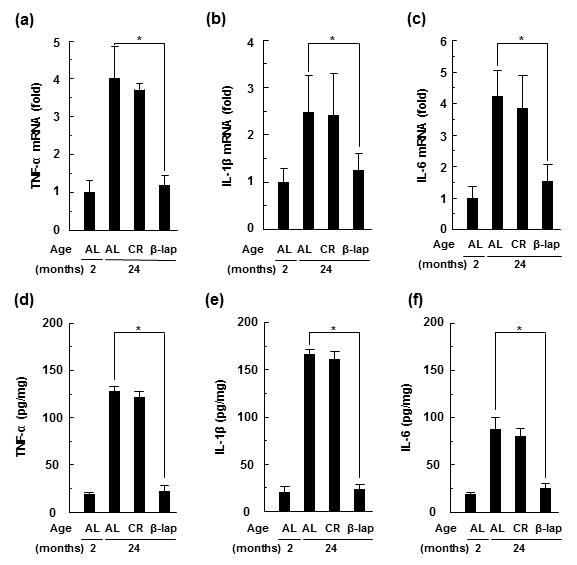

Supplement: Supplementary file 4 [file ACEL-18-e13016-s004.tif]

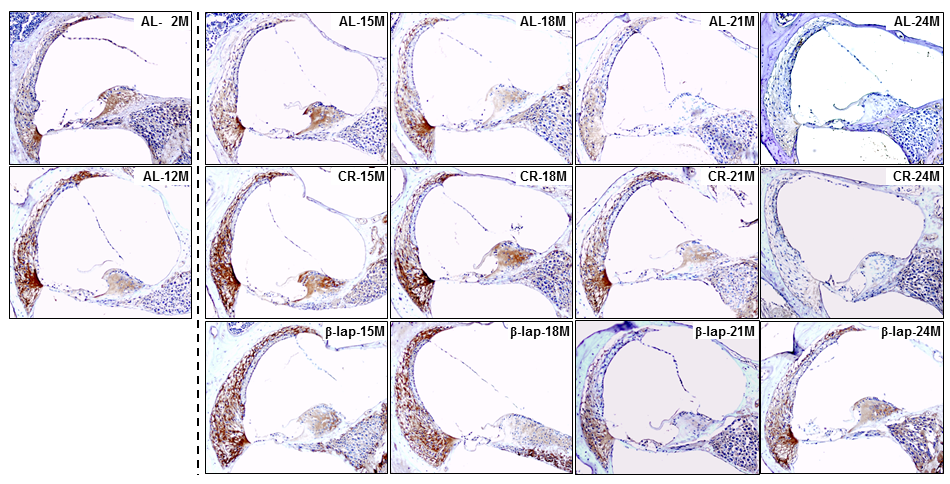

Supplement: Supplementary file 5 [file ACEL-18-e13016-s005.tif]
