# Supplementary material for: Augmentation of cellular NAD+ by NQO1 enzymatic action improves age‐related hearing impairment
Source: Aging Cell. 2019 Jul 28;18(5):e13016. doi: 10.1111/acel.13016 (PMC6718544; doi:10.1111/acel.13016)
Supplement: Supplementary file 6 [file ACEL-18-e13016-s006.docx]

**Supporting information listing**

**EXPERIMENTAL PROCEDURES**

**Table S1** Summary of immunohistochemical intensities for inflammatory and ROS production related mediators.

**Figure S1** β-lap markedly reduced age-related cochlear damages.

**Figure S2** Immunohistochemical staining of a typical inflammatory cytokine, TNF-α, in mouse cochleae.

**Figure S3** Immunohistochemical staining of 8-hydroxyguanosin in mouse cochleae.

**Figure S4** β-lap attenuated expression of pro-inflammatory cytokines.

**Figure S5** Immunohistochemical staining for SIRT1 in mouse cochleae.

**EXPERIMENTAL PROCEDURES**

**Reagents.** β-lap was chemically synthesized by ERUM Biotechnologies Inc. (Suwon, Korea) and micronized as particles coated with calcium silicate to enhance oral bioavailability. Dicoumarol was purchased from Calbiochem (San Diego, CA, USA). ABT-888 was purchased from Enzo Life Sciences (Plymouth, PA, USA). Anti-HMGB1 antibody was purchased from Sigma Chemical Co (St. Louis, MO, USA). Recombinant mouse TNF-α, IL-1β, IL-6, and antibodies against TNF-α, IL-1β, and IL-6 were purchased from R&D Systems Inc (Minneapolis, MN, USA). Antibodies to p-IκBα, acetyl-NF-κB p65, acetyl-p53 and p53 were purchased from Cell Signaling Inc. (Beverly, MA, USA). Antibodies against NF-κB p65, NQO1, SIRT1, and SIRT3 were purchased from Santa Cruz Biotech Inc. (Santa Cruz, CA, USA). DMEM, FBS, and other tissue culture reagents were obtained from Life Technologies Inc. (Gaithersburg, MD, USA).

**Cell culture**. The establishment and characterization of conditionally immortalized HEI-OC1 auditory cells has been described in our previous report1. Expression of outer hair cell specific markers such as Math1 and Myosin 7a suggests that HEI-OC1 cells represent OHC precursors. HEI-OC1 cells were maintained in high glucose DMEM containing 10% FBS. HEI-OC1 cells (5 x 104 cells/well on 24-well plates) were incubated with cytokine mixture (CM, consisting of 20ng/ml of each of TNF-α, IL-1β, and IL-6) in the presence of varying concentrations of β-lap

**Animals**. C57BL/6 male mice were obtained from the Central Laboratory Animal Inc. (Seoul, Korea). The animals were acclimatized to a 12 h light-dark cycle at 23 ± 1 ºC for 12 months with free access to food and water. All animal studies were approved by the Animal Care and Use Committee at Wonkwang University School of Medicine.

**Measurement of ABR**. The auditory thresholds were measured every 3 months from 12 to 24 months of age. The auditory thresholds were measured in response to tone bursts of 4, 8, 16 and 32 kHz (1 ms rise/fall time, 2 ms plateau) that were generated via an ABR workstation (Tucker-Davis Technologies, FL). Mice were anesthetized using a cocktail of ketamine (40 mg/kg) and xylazine (10 mg/kg) and kept warm with a heating pad during ABR recording. A subdermal (active) needle electrode was inserted at the vertex, while ground and reference electrodes were inserted subdermally in the loose skin beneath the pinnae of opposite ears. The ABR waveforms were averaged over a 10-ms time window, using Tucker-Davis ABR workstation software. The sound intensity was varied at 10-dB intervals near the threshold. Judgment of the threshold was made off-line by 2 independent, experimentally blind observers based on the ABR records.

**Transfection with siRNAs constructs**. Predesigned siRNAs against mouse NQO1 and control scrambled siRNA were purchased from Santa Cruz Biotechnology. The NQO1 siRNAs construct is a pool of 3 sequences of siRNA as follows: Duplex 1 Sense Strand: 5’-GGA UGA UGA GAG AAA CAA A-3’, Duplex 2 Sense Strand: 5’- CUC GCU GCA UGA ACU AUG A-3’, Duplex 3 Sense Strand: 5’- GAA UCC AGA CAU UGU UUC A-3’. The SIRT1 siRNAs construct is a pool of 3 sequences of siRNA as follows: Duplex 1 Sense Strand: 5’- CAU CUU GCC UGA UUU GUA ATT -3’, Duplex 2 Sense Strand: 5’- GUA CCA CCA AAU CGU UAC ATT -3’, Duplex 3 Sense Strand: 5’- GCA UAG AUC UUC ACC ACA ATT -3’. The SIRT3 siRNAs construct is a pool of 3 sequences of siRNA as follows: Duplex 1 Sense Strand: 5’- CCA AUG UCA CUC ACU ACU UTT -3’, Duplex 2 Sense Strand: 5’- CAA GGU UCC UAC UCC AUA UTT -3’, Duplex 3 Sense Strand: 5’- CCA ACA CGU UUA CAA ACA UTT -3’. The cells were transiently transfected with 100 nM siRNA constructs in X-tremeGENE siRNA transfection reagent (Roche Applied Science, Penzberg, Germany), according to the manufacturer’s protocol. The interference of expression was confirmed by immunoblot analysis.

**Measurement of pro-inflammatory cytokines**. To measure the levels of TNF-α, IL-1β and IL-6, cochlear tissues were extracted from mice following sacrifice and stored at −80 °C until use. Thereafter, the levels of TNF-α, IL-1β and IL-6 were determined by each ELISA kit (ELISA, Quantikine Kit; R&D Systems, Minneapolis, MN, USA) according to the manufacturer’s instructions.

**Measurement of NAD^+^ contents**. NAD^+^ contents were measured using a fluorescent NAD^+^ detection kit (Cell Technology Inc., Mountain View, CA, USA) following the manufacturer's instruction. Briefly, cochlear tissues were homogenized in 200 μl acidic extraction buffer to measure cellular NAD^+^ contents. Homogenates were then neutralized, and after an enzymatic cycling reaction, the NAD^+^ contents were measured using a fluorescence microplate reader.

**Measurement of SIRT1 and SIRT3 activity**. Experiments testing the effects of a CM and β-lap on SIRT1 and SIRT3 activity were performed using a fluorescent SIRT1 and SIRT3 assay kit (Enzo Life Sciences International Inc., PA) according to the manufacturer’s instruction. Briefly, the SIRT1 activity assays were performed using Fluor de Lys-SIRT1 as the substrate in SIRT1 assay buffer (25 mM Tris-Cl, pH 8.0, 137 mM NaCl, 2.7 mM KCl, 1 mM MgCl2, 1 mg/ml bovine serum albumin), and the Sirt3 activity assays were performed using Fluor de Lys-SIRT3 as the substrate in SIRT3 assay buffer (50 mM Tris-Cl, pH 8.0, 137 mM NaCl, 2.7 mM KCl, 1 mM MgCl_2_, 1 mg/ml bovine serum albumin) in a 96-well plate. Reactions were initiated by adding each substrate solution. After incubation at 37 ^o^C for 1 h, the plate was further incubated with developing solution for 10 min. Fluorescence readings were obtained using the CytoFluor series 4000 fluorometer (Perseptive Biosystems Inc., Framingham, MA) with the excitation wavelength set to 360 nm and the emission set to 460 nm.

**Measurement of NF-κB activity.** Cells were transiently transfected with NF-κB luciferase reporter plasmid using the transfection reagent, Lipofectamine 2000. After 24 h of incubation, the cells were treated with CM for 24 h in the presence of β-lap. The cells were then washed twice with PBS buffer and subsequently lysed in reporter lysis buffer (Promega, Madison, WI). A 20 μl aliquot of the lysate was mixed with 100 μl of luciferase assay reagent, and the emitted light intensity was measured using a luminometer AutoLumat LB953 (EG and G Berthold, Bad Wildbad, Germany). Finally, the luciferase activity was measured in triplicate, averaged, and then normalized against the β-galactosidase activity using the galactosidase assay system (Galacto-Light, Tropix Inc., MA) according to the manufacturer’s instruction.

**Measurement of PARP-1 activity.** PARP activity was assayed using the Universal Chemiluminescent PARP Assay Kit (Trevigen, Gaithersburg, MD, USA) following the manufacturer’s instructions. The lysate (30μg/well) were added into the wells containing PARP buffer cocktail, and then incubated at room temperature for 1 h. All wells were washed thrice with PBS plus 0.1% Triton X-100 (PBST), followed via incubation with a streptavidin-horseradish peroxidase in strep diluent buffer (1:1000 dilution) for 1 h. After extensively washes with PBST, chemiluminescent detection was performed. The background readings were subtracted from the readings of samples, and PARP activity was analyzed using a standard curve.

**Analysis of ATP Concentrations.** Intracellular ATP levels were measured using EnzyLight^TM^ ATP assay (BioAssay Systems, Hayward, CA, USA) according to the manufacturer's instructions. Luminescence was measured on a luminometer AutoLumat LB953 (EG and G Berthold, Bad Wildbad, Germany) and quantitated to ATP standards.

**Measurement of mitochondrial ETC complex I activity**. The enzymatic activity of complex I was assayed using the recently developed sensitive spectrophotometric method3. In brief, 50 μg (20μl) of mitochondrial proteins were suspended in 960 μl of medium containing 20 mM KH2PO4, 3.5 g/L BSA, 60 μM 2,6-dichloroindophenol, 70 μM decylubiquinone, 1 μM antimycin-A at 30 °C. After 3 min, 20 μl of 10 mM NADH was added and the absorbance was measured at 30-s intervals for 4 min at 37 °C. After that, 1 μl rotenone (1 mM in DMSO) was immediately added and the absorbance was measured again at 30-s intervals for an additional 4 min in order to confirm the presence of the rotenone-sensitive complex I. Complex I activity was expressed as U/g protein, in which 1 U complex I activity equals a reduction of 1 μmol DCIP per min.

**Preparation of subcellular fraction**. Cells were washed with ice-cold PBS, scraped and centrifuged at 1000×g for 5 min at 4 °C. The cell pellet was then resuspended in 200 μl of lysis buffer (10 mM HEPES, pH 7.9, 1.5 mM MgCl2, 10 mM KCl, 0.5 mM phenylmethylsulfonyl fluoride, and 0.5 mM dithiothreitol), and then incubated on ice for 15 min. At the end of incubation, 10 μl of 10% NP-40 were added and the tube was vortexed for 10 s. After centrifugation at 13,000×g for 1 min at 4 °C, the supernatant (cytosolic extract) was collected and stored at −80°C, while the pellet was further processed to obtain the nuclear extracts. The pellet was then resuspended in extraction buffer (5 mM HEPES, pH 7.9, 1.5 mM MgCl_2_, 0.5 mM phenylmethylsulfonyl fluoride, 0.2 mM EDTA, 0.5 mM dithiothreitol and 25% (vol/vol) glycerol) and incubated for 30 min at 4°C. Nuclear extracts were isolated by centrifugation at 13,000×g for 30 min at 4°C. The supernatant was then removed and stored at -80°C until used for western blot analysis. The mitochondrial fraction was purified using a mitochondria isolation kit (Sigma-Aldrich Inc., CA). Briefly, cells were harvested, centrifuged at 500×g for 5 min at 4 °C, and resuspended in extraction buffer A (10 mM HEPES, pH 7.5, 200 mM mannitol, 70 mM sucrose and 1mM EGTA). Cell disruption was performed by passing the cells through a 23-gauge needle 3-5 times. The homogenates were spun at 600×g for 5 min at 4°C. The supernatants were removed and spun at 11,000×g for 10 min at 4°C. The mitochondrial pellets were resuspended with storage buffer (10 mM HEPES, pH 7.4, 250 mM sucrose, 5 mM sodium succinate, 2 mM K2HPO4, and 1 mM DTT). Finally, the protein concentration was determined by the Lowry method.

**Western blot analysis**. Total proteins from HEI-OC1 and MEF cells were extracted in ice-cold lysis buffer, and the contents were measured using the Bio-Rad protein assay kit (Bio-Rad Laboratories, Hercules, CA). Twenty micrograms of protein were then subjected to electrophoresis on 10% SDS-polyacrylamide gels for 3 h at 20 mA, after which the protein was transferred to a nitrocellulose membrane. The membrane was then incubated in 5% (wt/vol) dried milk protein in PBS containing 0.05% (vol/vol) Tween-20 (PBS-T) for 1 h, after which was washed in PBS-T, and then further reacted with primary antibody (1:1,000) for 1 h. Next, the membrane was extensively washed with PBS-T and incubated with the appropriate secondary antibodies for 1 h at room temperature. After extensive washes, protein bands on the membrane were visualized using chemiluminescent reagents according to the manufacturer’s instructions (Supersignal Substrate; Pierce, Rockford, IL).

**Quantitative Real-time PCR (qRT-PCR) analysis**. Total RNA was isolated using TRIzol (Invitrogen, CA, USA) according to the manufacturer’s protocol. Three micrograms of RNA were converted into cDNA using the First Strand cDNA Synthesis Superscript kit (Invitrogen) according to the manufacturer’s protocol. Quantitative real-time PCR was performed using SYBR Green (Invitrogen). Reactions were performed in triplicate and the specificity was monitored using melting curve analysis after cycling. The primers used were as follows: Mitochondrial DNA, 5′-CCG CAA GGG AAA GAT GAA AGA C-3′ and 5′-TCG TTT GGT TTC GGG GTT TC-3′; Nuclear DNA, 5′-GCC AGC CTC TCC TGA TTT TAG TGT-3′ and 5′-GGG AAC ACA AAA GAC CTC TTC TGG-3′; TFAm, 5'-CCA AAA AGA CCT CGT TCA GC-3' and 5'-ATG TCT CCG GAT CGT TTC AC-3'; PGC-1α, 5′-AAG TGT GGA ACT CTC TGG AAC TG-3' and 5'-TGC TGC TGT CCT CAA ATA CG-3'; SIRT1, 5′-CAG TGT CAT GGT TCC TTT GC-3′, and 5′- CAC CGA GGA ACT ACC TGA T -3′; GAPDH, 5′-TCC CAC TCT TCC ACC TTC GA-3′ and 5′-AGT TGG GAT AGG GCC TCT CTT G-3′. Relative mRNA expression was quantified using the ΔΔCt method and GAPDH was used as an internal control. The results were expressed as fold change.

**Measurement of microRNAs expression**. For evaluation of microRNA expression, total RNA including the small RNA fraction was extracted from isolated cochlea and HEI-OC1 cells using a mirVana miRNA isolation kit (Ambion, Austin, TX, USA) following the manufacturer's instructions. Extracted RNA was reverse transcribed by using Taqman miRNA Reverse Transcription Kit and then amplified in a 20 μl PCR and primer set for amplification of miR-34a (Assay ID: 000426), miR-181 (Assay ID: 000480), miR-9 (Assay ID: 000583), miR-146 (Assay ID: 000468) and U6 (Assay ID: 001093) using TaqMan miRNA assays following the manufacturer's recommended protocol. The amplification step consisted of an initial denaturation at 95 °C, followed by 40 cycles of denaturation at 95 °C for 15 s and then annealing at 60 °C for 1 min. All reactions were carried out on the LightCycler PCR system (Roche Applied Science) using the TaqMan Universal PCR master Mix. Relative levels were determined using the ΔΔCt (threshold cycle value) method with U6 as an endogenous control, and the fold changes were calculated for each sample.

**Immunohistochemical staining and TUNEL assay**. The removed temporal bone was fixed in 4% paraformaldehyde for 16 h and then decalcified with 10% EDTA in PBS for 2 weeks, after which it was dehydrated and embedded in paraffin wax. Next, 5-µm thick sections were deparaffinized in xylene and rehydrated through graded concentrations of ethanol. For the immunohistochemistry study, an immunohistochemistry kit (DAKO LSAB Universal K680, Carpinteria, CA) was used and procedures were conducted according to the manufacturer’s instructions. The endogenous peroxidase was then blocked with 3% hydrogen peroxide for 5 min at room temperature. After the sections were washed in PBS, nonspecific binding was blocked with 1% bovine serum albumin for 1 h. Primary antibodies were then added to the slides, after which the incubation proceeded for 1 h. After repeated washes with PBS, the sections were incubated with biotinylated secondary antibody for 30 min and then covered with streptavidin-peroxidase for 15 min. Finally, the sections were stained in a freshly prepared substrate solution (3 mg of 3-amino-9-ethylcarbazole in 10 ml of sodium acetate buffer (pH 4.9), 500 µl of dimethylformamide, 0.03 % hydrogen peroxide) for 5 min. The nuclei of the immuno-stained cells were then counterstained with Mayer’s hematoxylin (Sigma-Aldrich Co.). Melanin pigment was visualized with the Fontan–Masson melanin staining method. Apoptotic cells were detected in situ using terminal deoxynucleotidyl transferase-mediated dUTP nick end-labeling (TUNEL) assay (TUNEL POD kit, Roche Molec Biochemic, Mannheim, Germany). Briefly, a section was deparaffinized and rehydrated. After incubation with 20 µg/ml proteinase K (Boehringer Mannheim, Mannheim, Germany), the endogenous peroxidase was blocked by incubating the samples in 2% H2O2 in methanol for 30 min at room temperature. Next, the tissue sections were washed in PBS and incubated with labeling solution for 1 h at 37°C. The nuclei were then counterstained with propidium iodide (0.5 μg/ml, Molecular Probes) for 10 min at RT. After washes with PBS, the specimens were examined under a fluorescence microscope.

**Electron microscopic evaluation.** The bulla was opened and the cochlea perfused via the oval and round windows with 2% glutaraldehyde in 0.1M phosphate buffer (pH 7.4). Fixation was continued by emersion for 2 h. The samples were decalcified in 10% EDTA on a rotator in a cold room for 3 days. EDTA was changed daily. Samples were washed in phosphate buffer and post-fixed in 1% OsO4 in phosphate buffer (pH 7.4) for 1 h. They were washed again in buffer, dehydrated in a graded series of ethanol, followed by propylene oxide, and embedded in epoxy resin. For electron microscopy, 70-nm-thick samples were cut, stained with uranyl acetate and lead citrate, and examined under a Tecnai G2 Spirit Twin transmission electron microscope (FEI Company, USA) and a JEM ARM 1300S high voltage electron microscope (JEOL, Japan).

**Statistical analysis.**

Results were expressed as mean ± SD. Differences between groups were examined for statistical significance using Student’s t test and analysis of variance (ANOVA). Values of p < 0.05 were considered statistically significant.

Table S1 Summary of immunohistochemical intensities for inflammatory and ROS production related mediators. The color intensity was arbitrarily scaled according to the intensity of TNF-α, as shown in supplementary Figure S2.

|  | **Cochlear Tissues** | **Age (months) of AL** | | | | | | **Age (months) of CR** | | | | **Age (months) of β-lap** | | | |
| --- | --- | --- | --- | --- | --- | --- | --- | --- | --- | --- | --- | --- | --- | --- | --- |
|  |  | **2** | **12** | **15** | **18** | **21** | **24** | **15** | **18** | **21** | **24** | **15** | **18** | **21** | **24** |
| **TNF-α** | **OC^a^**  **SG**  **SL**  **SV** | **-**  **-**  **-**  **-** | **-**  **-**  **-**  **-** | **-**  **+**  **++**  **+** | **+**  **+++**  **++**  **+** | **+**  **+**  **++**  **+** | **++**  **++**  **+++**  **++** | **-**  **-**  **-**  **-** | **-**  **+**  **+**  **-** | **+**  **+**  **++**  **+** | **++**  **++**  **+++**  **+** | **-**  **-**  **-**  **-** | **-**  **-**  **-**  **-** | **-**  **-**  **-**  **-** | **-**  **+**  **+**  **+** |
| **IL-1β** | **OC**  **SG**  **SL**  **SV** | **-**  **-**  **-**  **-** | **-**  **-**  **-**  **-** | **-**  **-**  **+**  **-** | **+**  **++**  **+++**  **+** | **-**  **++**  **++**  **+** | **-**  **++**  **+++**  **++** | **-**  **-**  **+**  **-** | **-**  **-**  **+**  **-** | **-**  **+**  **++**  **-** | **+**  **++**  **+++**  **+** | **-**  **-**  **+**  **-** | **-**  **+**  **+**  **-** | **-**  **+**  **+**  **-** | **-**  **++**  **+**  **-** |
| **IL-6** | **OC**  **SG**  **SL**  **SV** | **-**  **-**  **-**  **-** | **-**  **-**  **-**  **-** | **-**  **-**  **-**  **-** | **-**  **-**  **-**  **-** | **-**  **-**  **-**  **+** | **+**  **+**  **++**  **+** | **-**  **-**  **-**  **-** | **-**  **-**  **-**  **-** | **-**  **-**  **-**  **+** | **+**  **+**  **+**  **+** | **-**  **-**  **-**  **-** | **-**  **-**  **-** | **-**  **-**  **-**  **-** | **+**  **+**  **+ -** |
| **NF-κB** | **OC**  **SG**  **SL**  **SV** | **+**  **+**  **+**  **+** | **+**  **+**  **++**  **++** | **+**  **+**  **++**  **+** | **+**  **++**  **++**  **+** | **+**  **+**  **++**  **+** | **+**  **++**  **+++**  **+** | **-**  **+**  **+**  **+** | **+**  **+**  **++**  **+** | **++**  **+**  **++**  **+** | **+**  **+**  **++**  **+** | **+**  **+**  **++**  **+** | **+**  **+**  **++**  **+** | **+**  **+**  **++**  **+** | **-**  **+**  **+**  **+** |
| **HMGB1** | **OC^a^**  **SG**  **SL**  **SV** | **-**  **+**  **+**  **-** | **-**  **+**  **+**  **+** | **++**  **+++**  **+++**  **++** | **++**  **+++**  **++**  **++** | **+++**  **+++**  **+++**  **++** | **+++**  **+++**  **++**  **++** | **-**  **+**  **+**  **-** | **+**  **+**  **++**  **+** | **++**  **++**  **+++**  **++** | **+**  **+**  **++**  **+** | **-**  **+**  **+**  **-** | **-**  **+**  **+++**  **-** | **-**  **+**  **+**  **-** | **-**  **+**  **+ +** |
| **AGE** | **OC**  **SG**  **SL**  **SV** | **+**  **+**  **+**  **+** | **++**  **+**  **+**  **+** | **++**  **++**  **++**  **+** | **+**  **+**  **+**  **+** | **++**  **+**  **+++**  **++** | **+++**  **+++**  **+++**  **+++** | **+**  **+**  **+**  **+** | **+**  **+**  **+**  **+** | **++**  **+**  **+++ +** | **+++**  **+++**  **+++**  **+++** | **++**  **+**  **+**  **+** | **+++**  **+++**  **++**  **+++** | **+**  **+**  **+**  **+** | **+**  **++**  **+**  **+** |
| **RAGE** | **OC**  **SG**  **SL**  **SV** | **-**  **-**  **-**  **-** | **-**  **-**  **-**  **-** | **+**  **+**  **+**  **+** | **+**  **+**  **++**  **++** | **+**  **+**  **++**  **+** | **++**  **++**  **+++ ++** | **+**  **+**  **+**  **-** | **-**  **+**  **+**  **-** | **+**  **+**  **++**  **-** | **+++**  **+++**  **+++**  **++** | **+**  **+**  **+**  **-** | **+**  **+**  **+**  **+** | **+**  **+**  **+**  **+** | **+**  **++**  **+**  **+** |
| **NOX3** | **OC**  **SG**  **SL**  **SV** | **+**  **+**  **+**  **+** | **+**  **+**  **++**  **++** | **++**  **+++**  **+++**  **++** | **+**  **++**  **+**  **+** | **++**  **+++**  **+++**  **++** | **+++**  **++**  **+++**  **++** | **-**  **+**  **+**  **+** | **+**  **+**  **++**  **+** | **+**  **+**  **+**  **+** | **++**  **+++**  **+++**  **+** | **+**  **+**  **++**  **+** | **+**  **+**  **++**  **-** | **+**  **+**  **+**  **-** | **+**  **+**  **+**  **-** |

**^a^OC, organ of corti.; SG, spiral ganglion; SL, spiral ligament; SV, stria vascularis**

Figure S1 β-lap markedly reduced age-related cochlear damages. Cochleae from C57BL/6J mice fed with AL, CR, and β-lap were removed and embedded in paraffin. Next, 5-μm-thick sections were TUNEL stained as described in "Materials and Methods" section and visualized under a fluorescent microscope. The TUNEL-positive nuclei were visualized as green. Counterstaining was conducted with propidium iodide and nuclei were visualized as red. OC: organ of Corti; SG: spiral ganglion neuron; SL: spiral ligament; SV: stria vascularis.

Figure S2 Immunohistochemical staining of a typical inflammatory cytokine, TNF-α, in mouse cochleae. Cochleae from C57BL/6 mice fed AL, a CR diet, and β-lap-supplemented diet were removed, decalcified, and embedded in paraffin. Next, 5-μm-thick sections were prepared. All immunohistochemical staining procedures are described in “Methods”. OC, organ of Corti; SG, spiral ganglion neuron; SL, spiral ligament; SV, stria vascularis.

Figure S3. Immunohistochemical staining of 8-hydroxyguanosin in mouse cochleae. Cochleae from C57BL/6J mice fed AL, a CR diet, or a β-lap-supplemented diet were removed, decalcified, and embedded in paraffin. Next, 5-μm-thick sections were prepared. All immunohistochemical staining procedures are described in “Materials and Methods” section. OC: organ of Corti; SG: spiral ganglion neuron; SL: spiral ligament; SV: stria vascularis.

Figure S4 β-lap attenuated expression of pro-inflammatory cytokines. (A-C) Cochlear mRNA levels of TNF-α (A), IL-1β (B), IL-6 (C) measured using qRT-PCR. (D-F) Cochlear protein levels of TNF-α (D), IL-1β (E), IL-6 (F) analyzed by ELISA. Data are means ± SD (N=5). *p < 0.05, **p < 0.01 vs. 24-month-old ad libitum-fed mice (AL group).

Figure S5 Immunohistochemical staining for SIRT1 in mouse cochleae. Cochleae from C57BL/6 mice fed AL, a CR diet, and β-lap-supplemented diet were removed, decalcified, and embedded in paraffin. Next, 5-μm-thick sections were prepared. All immunohistochemical staining procedures are described in “Methods”. OC, organ of
